# A comparative evaluation of penetration depth and surface microhardness of Resin Infiltrant, CPP-ACPF and Novamin on enamel demineralization after banding: an *in vitro* study

**DOI:** 10.1080/26415275.2021.1919119

**Published:** 2021-06-11

**Authors:** Nishita Rana, Namita Singh, Shaila, Abi. M. Thomas, Rajan Jairath

**Affiliations:** a Department of Pedodontics and Preventive Dentistry, Christian Dental College, Ludhiana, Punjab; b Department of Orthodontics and Dento-facial Orthopaedics, Christian Dental College, Ludhiana, Punjab

**Keywords:** CCP-ACPF, inhibit caries

## Abstract

**Background:**

The field of dentistry has been revolutionized by various concepts. Minimal invasive dentistry is the preferred treatment approach in the present era; therefore, various techniques have been advocated to arrest caries lesions at an early stage on the grounds of better understanding of the dynamic nature of dental caries. Thus, study was conducted to compare and evaluate the penetration depth and enamel microhardness of Resin Infiltrant, CPP-ACPF and Novamin on artificial demineralized enamel surface after orthodontic banding.

**Material and methods:**

Eighty extracted sound premolars were banded. The bands were cemented with type 1 GIC and further divided into – Group I: Resin Infiltrant, Group II: CCP-ACPF, Group III: Novamin and Group IV: Control. The samples were incubated for 30 days and then thermocycled. A window of 4 mm × 4 mm was prepared on the buccal surface of samples and artificially demineralized for 4 weeks. A single application was made for Resin Infiltrant, while Novamin and CCP-ACPF were applied twice daily. These samples were otherwise immersed in artificial saliva, and this was protocol was observed for 14 days. For the evaluation of penetration depth, 10 samples from each group were bucco-lingually sectioned and immersed in methylene blue dye solution for 24 h and then evaluated under stereomicroscope. For the enamel surface microhardness, remaining 10 samples from each group were embedded in acrylic resin with outer buccal surface exposed and were tested by a using digital Micro-Vickers hardness tester.

**Results:**

All groups showed a significant difference in the depth of dye penetration and surface microhardness compared to the control group. As compared to the other tested groups, Resin Infiltrant exhibited the highest significant reduction in demineralization and increased microhardness. Novamin had a deeper penetration and increase in microhardness as compared to CCP-ACPF.

**Conclusion:**

Resin Infiltrant exhibited the highest potential to impede caries and constitutes a competent aerosol free micro-invasive strategy for combating non cavitated lesions approaching the outer layer dentine which are too advanced for remineralizing agents but do not necessarily require any drilling of tooth.

## 1. Introduction

*Dental caries* is a major public health issue that has affected children and adults universally. The latest report of Global Burden of Disease revealed that untreated dental caries remains the most common human disease condition worldwide. It is estimated that 2.3 billion people suffer from caries of permanent teeth and more than 530 million children suffer from caries of primary teeth [1]. White spot lesions (WSLs) are the first clinical sign of dental caries, with some subsurface mineral dissolution under intact enamel surface layer [2].

Stainless steel bands are an integral part of preventive and interceptive orthodontics that are extensively used in the field of paediatric dentistry. Because of the posterior position, there is a higher accumulation of plaque, exacerbating enamel decalcification around bands than brackets [3]. The ecological shift of cariogenic bacteria has been in orthodontic patients with higher levels of plaque than in non-orthodontic patients, resulting in more rapid caries progression than those without fixed appliances [4].

Literature validates that demineralized lesions can develop at a depth of 75 microns within four weeks during fixed orthodontic therapy and that 24% of untreated WSLs leads to cavitation [5]. Besides, children are more vulnerable to early decalcification of teeth because of insufficient oral hygiene and significantly higher critical pH than adults [6]. Despite various preventive measures, incipient lesions adjacent to orthodontic appliances continues to be the biggest challenge for the clinician.

The world of dentistry has seen a quantum shift with various new concepts and technologies, thereby setting high expectations for the longevity of teeth. In today’s scenario, radical ‘extension for prevention’ is an obsolete concept and has been substituted by non-invasive and minimal interventions to manage early lesions [7]. Hence, the goal is to reverse or arrest the progression of the lesion.

Remineralization is the cornerstone of non-invasive strategies of incipient lesions. One approach that enhances the anti-caries efficacy of fluoride is CPP-ACPF, designed to stabilize amorphous nano-complexes; that serves as a calcium and phosphate reservoir to facilitate remineralization. The incorporation of fluoride to CPP-ACP could give a synergistic effect [8]. Novamin is another non-fluoridated technology that immediately begins surface ion exchange, network disintegration of SiO2, and precipitation of calcium and phosphate to form an apatite layer in the presence of an aqueous medium [9]. However, dependence on home-based remineralizing agents is sometimes considered insufficient in subsurface defects, as they do not give immediate results. Besides, healing occurs superficially as the relatively thick, intact surface layer with high mineral content has been suspected of inhibiting remineralization while the body of the lesion remains porous. Thus, the caries process advances and encompasses the outer layer of dentin without showing any evident sign of cavitation [10,11].

Paris and co-workers in 2009 developed a new micro-invasive concept, Resin Infiltration, credited for being able to terminate the progression of early enamel lesions by occluding the micro-porosities within the lesion and preserving the integrity of the healthy tooth substrate. This novel technique narrows the difference between non-invasive and invasive methods for arresting the early non-cavitated lesions [12,13].

Since there is no definitive consensus on how to treat the early enamel lesions, this study aimed to compare the penetration depth and enamel microhardness of Resin Infiltrant with the tooth remineralizing agents CPP-ACPF and Novamin on demineralized enamel after orthodontic banding. The hypothesis tested was that there would be no difference in the penetration depth and enamel microhardness of the three different treatments.

## 2. Materials and methods

### 2.1. Sample collection

Eighty extracted caries-free permanent premolars indicated for orthodontic extraction were included in this study. Teeth with cracks, fluorosis, hypoplasia caries or restorations were excluded. Each tooth was thoroughly scaled to remove calculus and then polished with a slurry of pumice and water on a slow-speed handpiece. The teeth were stored in 0.1% thymol until use.

### 2.2. Banding

Customized stainless steel bands (0.150″×0.004″) were adapted and cemented around all the samples using type-1 glass ionomer cement. The eighty banded samples were divided into four groups (twenty each), namely – Group I: Resin Infiltrant (Icon Infiltrant, DMG, Hamburg, Germany), Group II: CCP-ACPF (GC Tooth Mousse Plus, GC Corp., Tokyo, Japan), Group III: Novamin (SHY-NM Toothpaste, Group Pharmaceuticals Ltd, Mumbai, India) and Group IV: Control (No treatment).

### 2.3. Incubation and thermocycling

The samples were transferred to four glass containers filled with deionized water and were incubated for 30 days at 37 °C to simulate cement dissolution in the oral cavity. Afterwards, the samples were thermocycled according to ISO:11405 protocol of 500 cycles between 5 °C and 50 °C with a dwell time of ≥20 s to replicate the temperature fluctuation of the oral cavity [14].

### 2.4. Artificial demineralization

Following thermocycling, the bands were removed, and enamel samples were prepared by coating with nail varnish and leaving a window of 4 mm × 4 mm on the buccal surfaces. The samples were immersed in Silverstone’s cariogenic solution (17% gelatine, 1 g/l synthetic hydroxyapatite and 0.1% thymol) for four weeks to induce artificial demineralization. The pH was adjusted to 4.3 by adding lactic acid. The solution was changed every week to avoid a potential fluoride build up in the solution [15,16].

### 2.5. Material application

Subsequent to the artificial demineralization, the samples were washed with deionized water before exposure to different surface treatments as stated below.

**Group I (Resin Infiltrant):** The samples were subjected to 15% HCl etching for 2 min, rinsing for 30 s, ethanol desiccation for 30 s and air-drying for 10 s, followed by application of Resin Infiltrant, which was allowed to penetrate for 3 min before being light-cured for 40 s using an LED light-curing unit. The infiltrated samples were then stored in artificial saliva for 14 days.

**Group II (CCP-ACPF):** CPP-ACPF containing tooth mousse was applied on samples using a cotton applicator for 3 min twice daily for 14 days, and they were preserved in artificial saliva.

**Group III (Novamin):** Novamin toothpaste was applied on samples using a cotton applicator for 3 min twice daily for 14 days and were preserved in artificial saliva

**Group IV (Control):** Samples were kept immersed in artificial saliva for 14 days.

### 2.6. Artificial saliva formulation

The treated samples were stored in artificial saliva at an ambient temperature. The artificial saliva was made using a formulation of 0.65 g/l potassium chloride, 0.058 g/l magnesium chloride, 0.165 g/l calcium chloride, 0.804 g/l dipotassium hydrogen phosphate, 0.365 g/l potassium dihydrogen phosphate, 2 g/l sodium carboxymethyl cellulose and distilled water. The pH was maintained at 7.2. The saliva was prepared and changed every daily for each treatment group.

### 2.7. Testing

#### 2.7.1. Penetration depth

Ten samples from each group were randomly chosen for the dye penetration test. The penetration depth of the materials into the lesions was determined by immersing the samples in methylene blue dye for 24 h at 37 °C and then sectioning the samples into two halves along the bucco-lingual plane with a diamond disc mounted on a low-speed handpiece. The sections were observed under a stereomicroscope at 40X magnification. The depth of dye penetration was measured in microns (µm) using Image Analysis Software (Motic Images plus 2.0 ML 2001–2004). The depth of penetration of the different test materials was assessed as the depth of dye penetration into the demineralized area of samples in groups I, II and III and was compared to the depth of demineralization of the non-treated teeth in control Group IV.

#### 2.7.2. Surface microhardness

The remaining ten samples from each group were decoronated at approximately 1 mm coronal to the cemento-enamel junction. The acrylic moulds were made using rubber pipe (diameter = 2.54 cm, height = 1.5 cm) .The crowns were embedded in acrylic blocks, leaving the buccal surfaces of the crown exposed, flat and parallel to the floor as required for microhardness testing. The surface microhardness was determined using a Micro-Vickers Hardness Tester (VHS 1000 A, SR 12050217, Banbros Engineering Pvt. Ltd.) with a Vickers elongated diamond pyramid indenter. A constant load of 100 g was applied to the surface for 10 s. Five indentations, at least 100 µm apart, were made at the centre of each sample. Digital readings were recorded for each indentation, and an average value of the Vickers hardness number (VHN) was calculated for each sample.

### 2.8. Statistical analysis

SPSS version 21.0. (Amonk, IBM Corp., NY) was used for statistical analysis of the data. The results were analysed by one-way analysis of variance (ANOVA) followed by unpaired t-tests. The level of significance was set at *p* < 0.05.

## 3. Results

### 3.1. Penetration depth

**Figure 1.**
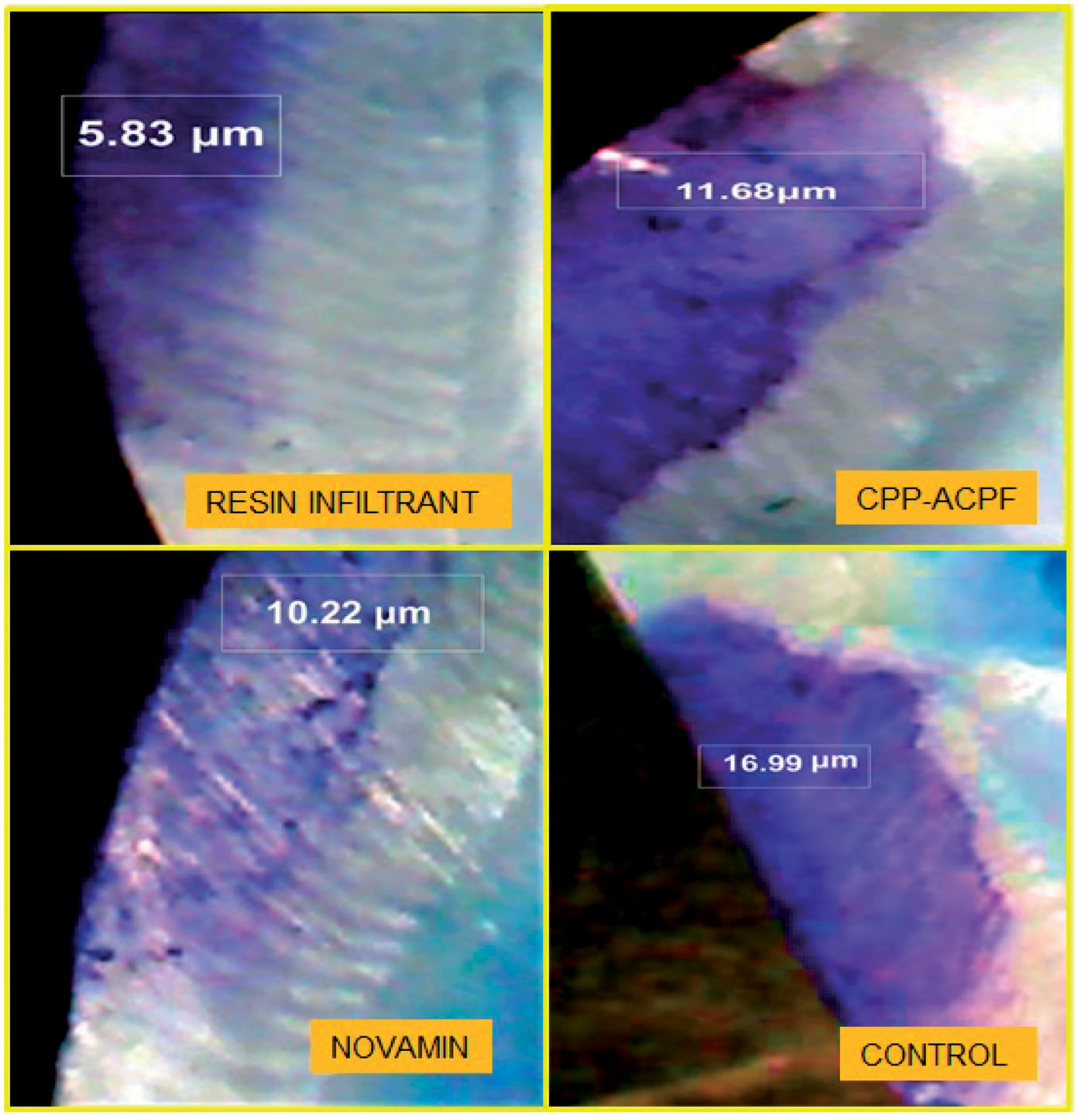
Dye penetration depth of different materials under study.

**Figure 2.**
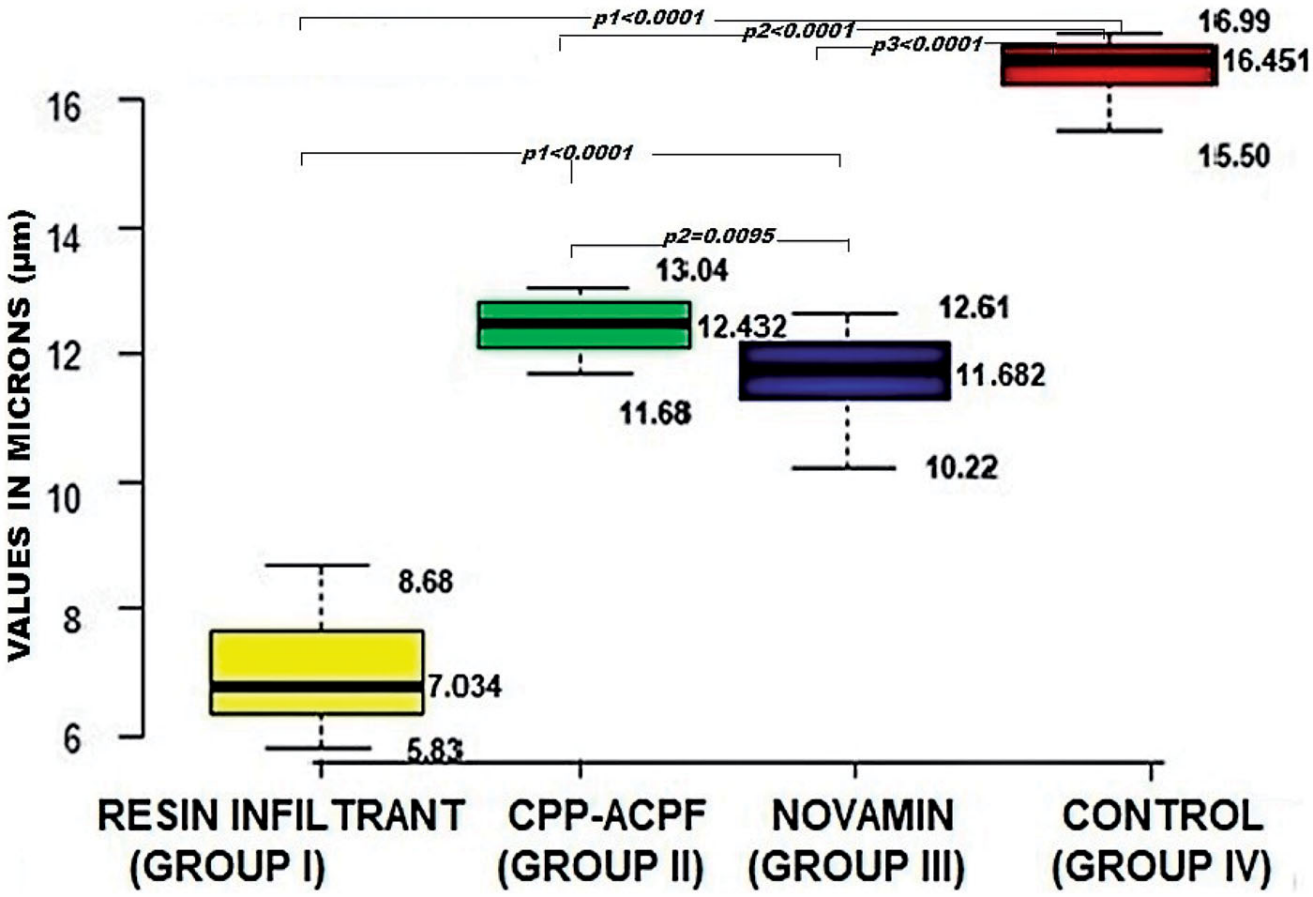
Box and Whisker plot showing minimum and maximum dye penetration depth in microns (μm).

The results of the dye penetration test are shown in Figures 1 and 2. The depths of dye penetration varied between 7.034 and 16.451 µm. The ANOVA showed a significant difference in the dye penetration depth between the four groups (*p* < 0.0001). The inter-group comparisons revealed that all three treatment groups had a significantly reduced dye penetration depth compared to the control group (*p*_1_, *p*_2_ and *p*_3_ < 0.0001). Furthermore, the Resin Infiltrant group showed the reduced depth of dye penetration compared to both CPP-ACPF and Novamin group (*p*_1_ < 0.0001). Finally, the Novamin group showed reduced dye penetration depth compared to the CPP-ACPF group (*p*2 = 0.0095). The order of reduction of lesion depth was Resin Infiltrant > Novamin > CPP-ACPF > Control. Hence, the null hypothesis was rejected.

### 3.2. Surface microhardness

**Figure 3.**
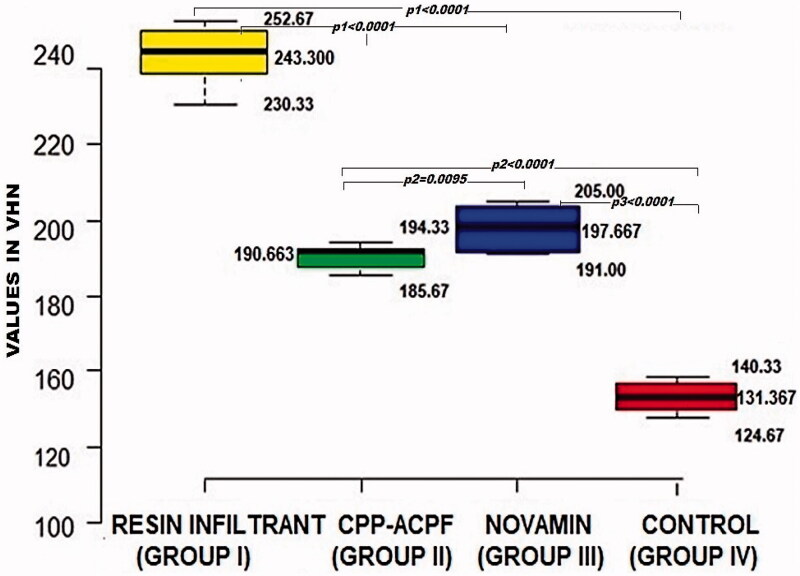
Box and Whisker plot showing maximum and minimum enamel surface microhardness in VHN

The results of the enamel microhardness measurements are shown in Figure 3. The miocrohardness varied between 131.367 and 243.300 VHN. The ANOVA showed a significant difference in microhardness between the four groups (*p* < 0.0001). The inter-group comparisons revealed that all three test material groups had a significantly higher surface microhardness compared to the control group (*p*_1_, *p*_2_ and *p*_3_ < 0.0001). Besides, the Resin Infiltrant group showed significantly highest surface microhardness than both CPP-ACPF and Novamin group (*p*_1_ < 0.0001). Lastly, the Novamin group showed higher surface microhardness than the CPP-ACPF group (*p*_2_ = 0.0095). The surface microhardness of enamel was in order Resin Infiltrant > Novamin > CPP-ACPF > Control. Hence, the null hypothesis was rejected

## 4. Discussion

Enamel decalcification adjacent to fixed orthodontic appliances is a predominant iatrogenic effect of orthodontic therapy. Enamel is an acellular tissue, and caries acts upon it through a chemical process. Thus, unlike other tissues, enamel cannot heal by cellular repair mechanism. It is a well-established fact that the formation of incipient lesions is a reversible process, where periods of demineralization alternates with periods of remineralization [17]. The caries prevention around orthodontic bands can be fortified using fluoride-releasing cements; however, these do not deter enamel demineralization under loose bands or at sites where cement has disintegrated. In a highly conducive environment, these lesions rapidly progress. If left untreated, they often develop into carious cavitations.

The contemporary field of dentistry has an upraised interest in minimally intrusive therapeutic procedures for dental caries, inspiring researchers to compare the effectiveness of various commercially available techniques. Thus, the present study was carried out to compare the caries inhibiting efficacy of new a Resin Infiltration technique with two conventional remineralizing agents CCP-ACPF and Novamin, as regards penetration depth of lesion and surface microhardness of enamel.

The penetration depth of a dye can be used to assess the penetration of materials into the demineralized enamel surface lesion. The estimated extent of dye penetration is comparable to the depth of the demineralized lesion. Therefore, material penetration is shown by an overall reduction in lesion depth [15,18]. Early enamel lesions have less microhardness than intact caries free enamel surfaces. Therefore, microhardness testing of demineralized and treated lesions has been done to obtain information about surface changes of enamel [19].

In the present study, the control group, consisting of enamel surfaces that had received no treatment, exhibited the highest dye penetration depth, whereas all three test materials showed a significant reduction in dye penetration depth. This indicates that there was significant penetration of the different test materials into the demineralized areas.

The Resin Infiltration technique used in this study consists of low viscosity resin with high penetration coefficient, which facilitated superior penetration into the lesion by capillary forces. Furthermore, the pseudo-hyper-mineralised layer’s inhibitory effect is eliminated by etching with 15% HCL, which erodes more than twice as much as phosphoric acid (58 µm), allowing dissemination into the deepest part of the lesion. As compared to resins without solvents, the use of 99% ethanol has been shown to improve the penetration coefficient by lowering contact angle. These material and procedural effects may have attribute to the significantly higher penetration depth of Resin Infiltrant into a lesion compared to other groups under study [20,21].

The present study’s findings come in agreement with those of Subramaniam et al. and Prajapati et al., who found that the infiltrant successfully penetrated into the artificially created lesion [18,19]. Congruently, Paris et al., in an *in vitro* study demonstrated significantly more penetration of Resin Infiltrant into pit and fissure lesions when compared to conventional sealants**. [**22] Kim et al. and Prasada et al. observed significant improvement in the shades of artificial lesions upon Resin Infiltrant application, accrediting greater penetration than other remineralizing agents [23,24]. Although, this was not the primary objective of the research, there was an increase in the hue of artificial lesions when treated with Resin Infiltrant. The fact that a separate protocol was employed in the present research for the formation of the artificial lesions.

Novamin showed significantly greater penetration than CPP-ACPF. The findings of this study were consistent with those of Lata et al., who found that fluoride and CPP-ACP had no additive remineralization potential and that fluoride, CPP-ACP, and their mixture were ineffective in remineralizing incipient lesions at the subsurface level [25]. Furthermore, unlike other calcium phosphate-based systems, ions released from Novamin directly frame hydroxyl-carbonate apatite (HCA) without the intermediate amorphous calcium phosphate (ACP) phase. The particles of Novamin precipitate and attach firmly to the tooth surface after the initial application. These deposits have been reported to show sustained release of ions for two weeks before transforming into HCA. This is perhaps one of the reasons for Novamin's superior penetration depth over CPP-ACPF [26].

The control group in this study exhibited the lowest mean microhardness indicating chemical breakdown of enamel rods, which causes voids and eventually softens the enamel. The significantly higher microhardness of the Resin Infiltrant may be credited to the competency of low-viscosity resin to seal the breaches between the remaining porous lesions and to create a diffusion barrier not just at the surface but also inside the enamel lesion body. The values for surface microhardness was in approximation to that of sound dental enamel. The incorporated resin matrix has been postulated to reinforce the tooth substance mechanically, besides hardness and fracture toughness of resins increase with the degree of conversion of double bonds. Hence, the demineralized tissues rehardens, and the mechanical characteristics of the tooth enhances [27].

The microhardness findings by Kim et al. and Paris et al. are similar to those of the current study [23,28]. When comparing enamel surfaces treated with an infiltrant to those with a fissure sealant, Taher et al. found that the infiltrant-treated enamel surfaces had significantly higher surface hardness [29]. Mandava et al. also demonstrated the better performance of esin nfiltration in recovering microhardness and penetrating deep into the porous artificial lesions compared to colloidal silica infiltrates [30]. Novamin had a significantly higher microhardness than CPP-ACPF in this study which was in agreement with the results of Raja et al. [31]. The high microhardness values for Novamin may be due to its larger and more angular deposits, which adhere to the tooth surface more compactly, as well as to the precipitation of ions that occlude dentinal tubules, unlike CCP-ACPF’s irregular and amorphous deposition [9,32].

In the current study, the penetration depth and surface microhardness of CPP-ACPF and Novamin were lower than those of Resin Infiltrant. This could be attributed to the reliance of remineralizing agents on the concentration gradient of Ca/p in saliva for the lesion apposition, which is a slow time-dependent process. The artificial saliva might have low Ca/p ions to provide a suitable concentration gradient, causing CPP-ACPF and HCA nano-complexes to precipitate insufficiently, and the body of the lesion to remain porous. , 14 days regimen used in the study could not completely remineralize artificial caries [33]. Similarly, Pancu et al. concluded that the caries esin Infiltration leads to an increase in the hardness of lesions compared to fluoride treatment, which depends on the treatment and the time of remineralization with artificial saliva [34].

As indicated by the results, all the materials used in study showed caries inhibiting potential. The Resin Infiltrant showed the highest penetration and maximum microhardness recovery of demineralized enamel immediately after a single application, whereas remineralizing agents required at least 14 days of regime. Thus Resin Infiltration is an effective micro-invasive technique that seem better suited to treat early caries lesions.

Resin Infiltration technology could be valuable to young patients. As it provides the early intervention of incipient caries, limiting the operative modalities and improving the patient’s acceptance to treatment and could, therefore, be incorporated regularly in clinical practice. Likewise, in the absence of good oral hygiene, it can be beneficial for patients undergoing fixed orthodontic therapy. Because of the excellent penetration ability, Resin Infiltrant can be used in non cavitated lesions too advanced for remineralizing agents but not requiring any tooth drilling. Besides, Resin Infiltrant has a refractive index closer to that of hydroxyapatite, thus improves the esthetic characteristics of white spot lesions.

*In vitro* studies have certain limitations like difficulty in simulating the oral environment. Further long-term studies and *in vivo* investigations are required to confirm the efficacy of preventive techniques under clinical conditions.

## 5. Conclusion

Resin Infiltrant exhibited the highest potential to impede caries. Therefore, it can be concluded that

1. The Resin Infiltration technique is a promising aerosol free strategy at the present time for combating advanced non-cavitated initial enamel lesions approximating dentine.
2. Novamin and CPP-ACPF can also be employed to the arrest early stages of enamel caries, particularly in those cases where patient compliance will not pose a hindrance.
